# FGF2 Stimulates COUP-TFII Expression via the MEK1/2 Pathway to Inhibit Osteoblast Differentiation in C3H10T1/2 Cells

**DOI:** 10.1371/journal.pone.0159234

**Published:** 2016-07-12

**Authors:** Mi Nam Lee, Jung-Woo Kim, Sin-Hye Oh, Byung-Chul Jeong, Yun-Chan Hwang, Jeong-Tae Koh

**Affiliations:** 1 Research Center for Biomineralization Disorders, School of Dentistry, Chonnam National University, Gwangju, Republic of Korea; 2 Department of Pharmacology and Dental Therapeutics, School of Dentistry, Chonnam National University, Gwangju, Republic of Korea; 3 Department of Conservative Dentistry, School of Dentistry, Chonnam National University, Gwangju, Republic of Korea; Kyungpook National University School of Medicine, REPUBLIC OF KOREA

## Abstract

Chicken ovalbumin upstream promoter transcription factor II (COUP-TFII) is an orphan nuclear receptor that regulates many key biological processes, including organ development and cell fate determination. Although the biological functions of COUP-TFII have been studied extensively, little is known about what regulates its gene expression, especially the role of inducible extracellular factors in triggering it. Here we report that COUP-TFII expression is regulated specifically by fibroblast growth factor 2 (FGF2), which mediates activation of the MEK1/2 pathway in mesenchymal lineage C3H10T1/2 cells. Although FGF2 treatment increased cell proliferation, the induction of COUP-TFII expression was dispensable. Instead, FGF2-primed cells in which COUP-TFII expression was induced showed a low potential for osteoblast differentiation, as evidenced by decreases in alkaline phosphatase activity and osteogenic marker gene expression. Reducing COUP-TFII by U0126 or siRNA against COUP-TFII prevented the anti-osteogenic effect of FGF2, indicating that COUP-TFII plays a key role in the FGF2-mediated determination of osteoblast differentiation capability. This report is the first to suggest that FGF2 is an extracellular inducer of COUP-TFII expression and may suppress the osteogenic potential of mesenchymal cells by inducing COUP-TFII expression prior to the onset of osteogenic differentiation.

## Introduction

Chicken ovalbumin upstream promoter-transcription factor II (COUP-TFII), also known as NR2F2, is an orphan nuclear receptor that plays key roles in cell fate determination and organ development [1–3]. COUP-TFII functions as a molecular switch for determining the specific lineage of mesenchymal cells; it acts as a negative regulator of osteoblast differentiation through inhibiting Runx2 function, whereas it acts as a positive regulator of both adipogenic and chondrogenic differentiation by inducing the expression of PPARγ and of Sox9, respectively [3, 4]. There is growing evidence in support of the important role of COUP-TFII in cell fate determination, whereas the regulatory mechanism of COUP-TFII expression has been elusive until now. Recent studies have shown that COUP-TFII expression is regulated by Shh, Wnt3a, miR-302a, and miR-194 during the differentiation process; however, it is restricted to adipogenesis and osteodifferentiation events [5–8]. Meanwhile, COUP-TFII levels were found to be high in uncommitted precursor cells but decreased in differentiated cells [3], suggesting that this nuclear receptor also plays a pivotal role in maintaining precursor cells. However, we still do not understand how COUP-TFII expression is regulated in undifferentiated cells and what intracellular signaling pathway is involved in COUP-TFII gene expression.

Growth factors, hormones, and morphogens regulate mesenchymal cell differentiation and cell maintenance. For example, bone morphogenetic proteins (BMPs) regulate both osteogenic and adipogenic differentiation [9, 10], and hepatocyte growth factors (HGFs) regulate cell proliferation and osteogenesis of precursor cells [11, 12]. The roles of transforming growth factors (TGFs), Wnts, and parathyroid hormones (PTHs) in osteogenic differentiation have been well studied [13, 14]. These factors stimulate intracellular signaling pathways, such as the Smads, MEK/ERK, and GSK/β-catenin pathways, which finally trigger specific gene expression to perform relevant cellular functions [9, 13]. Given the importance of COUP-TFII in determining the fate of precursor cells and in maintaining pluripotency of embryonic stem cells [3, 15, 16], it is necessary to understand what factors regulate COUP-TFII expression and which intracellular signaling pathways are linked to its expression in undifferentiated precursor cells.

In this study, we evaluated the effects of short-term treatment with several extracellular factors on COUP-TFII expression and mesenchymal cell properties. Among the various members of the BMP and FGF families, BMP2 and FGF2, respectively, were used as representative factors of the families, because BMP2 and FGF2 have been well characterized in terms of functioning and signaling mechanisms. Our results showed that FGF2 specifically induces the expression of COUP-TFII by MEK1/MEK2 pathway activation, suggesting that COUP-TFII acts downstream of this signaling pathway. Before the onset of differentiation, FGF2 priming (pre-exposure to FGF2) had a negative effect on osteogenic differentiation owing to greater COUP-TFII expression. Our study suggests for the first time that COUP-TFII expression can be regulated by FGF2 and that the timing of FGF2 treatment is an important factor in determining the lineage-specific differentiation of mesenchymal stem cells.

## Materials and Methods

### Cell culture and treatments

C3H10T1/2 and 3T3-L1 cells, obtained from the American Type Culture Collection (ATCC, Manassas, VA, USA), were maintained in Dulbecco’s modified Eagle’s medium (DMEM) (Gibco/Thermo Fisher Scientific, Waltham, MA, USA) containing 10% fetal bovine serum (FBS) (Gibco/Thermo Fisher Scientific), supplemented with 100 U/mL of penicillin and 100 μg/mL of streptomycin (Invitrogen/Thermo Fisher Scientific). MC3T3-E1 cells obtained from the ATCC were cultured in α-minimal essential medium (α-MEM) (Gibco/Thermo Fisher Scientific) supplemented with 10% FBS (Gibco/Thermo Fisher Scientific), 100 U/mL of penicillin, and 100 μg/mL of streptomycin (Invitrogen/Thermo Fisher Scientific). For ligand treatments, cells were seeded on culture plates (1×10^5^ cells/well in a 6-well plate) with growth culture media. After 24 h, the cells were changed with 0.1% FBS or 2% FBS-containing media and incubated for 18 h. The cells were then treated with the indicated amounts of recombinant mouse FGF2 (R&D Systems, Minneapolis, MN, USA), recombinant mouse HGF (R&D Systems), recombinant human bone morphogenetic protein 2 (BMP2) (CowellMedi, Busan, South Korea), human parathyroid hormone N-terminal active peptide fragment 1–34 (PTH) (Merck, Darmstadt, Germany), and insulin-like growth factor 1 (IGF1) (R&D Systems) in 0.1% FBS or 2% FBS-containing media for 24 h. For treatment with chemical inhibitors, the cells were pre-treated with the indicated amount of chemical inhibitors (5 μM of U0126, 10 μM of PD98059, 5 μM of LY294002, 10 μM of SP600125, and 10 μM of SB202190) (Sigma-Aldrich, St. Louis, MO, USA) for 30 min. The cells were then co-treated with FGF2 and the indicated chemical inhibitor in 2% FBS-containing media for 24 h. Treatment with 0.1% dimethyl sulfoxide (DMSO) served as a negative control. After treatment, cells were harvested and subjected to real-time reverse transcription polymerase chain reaction (RT-PCR) and immunoblot analysis.

### Transient transfection and viral infection

Cells were transfected transiently with specific siRNAs to knock down endogenous COUP-TFII, MEK2, cyclin D1, and c-Jun using Lipofectamine RNAiMAX (Invitrogen/Thermo Fisher Scientific). After 4 h of incubation with the siRNAs–liposome complex, the cells were changed with 2% FBS-containing media and cultured for 24 h. The cells were then treated with FGF2 with or without the indicated chemical inhibitors for 24 h. Pre-designed and validated siRNAs were used as follows: COUP-TFII Silencer Pre-designed siRNA (ID#139842, CAT# AM16708; Ambion/Thermo Fisher Scientific). The specificity and efficacy of this siRNA was validated in a previous study [17]. Knockdown of MEK2 was accomplished using two distinct non-overlapping siRNAs against MEK2, si-MEK2 #1 (AccuTarget siRNA, #1383748 Duplex; Bioneer, Daejeon, South Korea) and si-MEK2 #2 (AccuTarget siRNA, #1383749, Duplex; Bioneer). We also used si-c-Jun (AccuTarget siRNA, #1375140, Duplex; Bioneer) and si-Cyclin D1 (AccuTarget siRNA, #1336074, Duplex; Bioneer). Si-Control nontargeting siRNA (AccuTarget negative control siRNA; Bioneer) was used as a negative control. For viral infection, cells were treated with an adenovirus encoding COUP-TFII (Ad-COUP-TFII) (provided by Dr. Kee-Sook Lee, Chonnam National University, Gwangju, South Korea) at the designated multiplicity of infection under serum-free conditions. Green fluorescent protein adenovirus (Ad-GFP; 50 multiplicity of infection) (Seoulin Corp., Seoul, Korea) was used as a control. After 4 h, an equivalent volume of medium containing 4% FBS was added, and cells were incubated for the time period indicated. Non-targeting control siRNA and Ad-GFP did not show any significant effect on cellular function.

### RT-PCR and real-time RT-PCR

Total RNA was isolated from cells using TRIzol reagent (Invitrogen/Thermo Fisher Scientific), and 1 μg of total RNA was reverse transcribed using a reverse transcription system (200 U of M-MLV, 0.5 mM of dNTP, 40 U of RNAsin, and 2 μL of random primer (100 μg/mL) (Promega, Madison, WI, USA), according to the manufacturer’s protocol. PCR was carried out for 28 cycles (at 95°C for 30 sec, 55°C for 30 sec, and 72°C for 30 sec) using 5 μL of cDNA (5 μg/mL), the GoTaq Green Master Mix (Promega), and the following primers (10 pmole each): COUP-TFII, 5′-GCTTTCCACATGGGCTACAT-3′ and 5′-TGCATGCAGCCTAACAACAT-3′; and β-actin, 5′-TGGATGGCTACGTACATGGCTGGG-3′, and 5′-TTCTTTGCAGCTCCTTCGTTGCCG-3′. The PCR product was analyzed by means of agarose gel electrophoresis. For quantitative real-time PCR, the StepOnePlus Real-Time PCR System (ABI, Abilene, TX, USA) was used. PCR was carried out in a final volume of 20 μL using 10 pmole of each primer (listed below), 5 μL of cDNA (5 ng/μL), and 10 μL of Power SYBR Green PCR Master Mix (ABI, Valencia, CA, USA). PCR was performed with a 5 min pre-incubation period at 95°C, followed by 40 cycles of 30 sec each at 95°C, 30 sec at 55°C, and 30 sec at 72°C. All reactions were run in triplicate and the expression levels of all mRNAs were normalized to the expression level of endogenous β-actin. Relative target gene expression was quantified using the comparative Ct method. The sequences of the primers used were as follows: for β-actin, 5′-GCCATCTCCTGCTCGAAGTC-3′ and 5′-ACCCACACTGTGCCCATCTA-3′; for COUP-TFII, 5′-AAGAGCTTTCCGAACCGTGTT-3′ and 5′-CGCTCCTTGCCGCTGCT-3′; for alkaline phosphatase (ALP), 5′-TTTCCCGTTCACCGTCCAC-3′ and 5′-ATCTTTGGTCTGGCTCCCATG-3′; for Osterix, 5′-GCGGCTGATTGGCTTCTTCT-3′ and 5′-AGCGACCACTTGAGCAAACA-3′; for Runx2, 5′-ATAGCGTGCTGCCATTCGAGGT-3′ and 5′-TCTCCAACCCACGAATGCACTA-3′; and for osteocalcin (Oc), 5′-GCTGTGACATCCATTACTTGC-3′ and 5′-CTCCTGAGTCTGACAAAGCCTT-3′; for BSP, 5′-CCTTGTAGTAGCTGTATTCATCCTC-3′ and 5′-AAGCAGCACCGTTGAGTATGG-3′.

### Immunoblot analysis

Cells were harvested and lysed with lysis buffer (Cell Signaling Technology, Danvers, MA, USA). Total protein was quantified using the BCA protein assay reagent (Bio-Rad Laboratories, Hercules, CA, USA). Proteins were resolved by 10% SDS-PAGE and transferred to a PVDF membrane. After blocking with Tris-buffered saline containing 0.1% Tween-20 (Bio-Rad Laboratories) and 5% skim milk (Gibco/Thermo Fisher Scientific), the membrane was incubated with primary antibodies against COUP-TFII (1:500, Abcam, Cambridge, MA, USA), MEK2 (1:2000, Cell Signaling Technology), Cyclin D1 (1:2000, Cell Signaling Technology), c-Jun (1:2000, Cell Signaling Technology), c-Fos (1:1000, Cell Signaling Technology), and β-actin (1:1000, Santa Cruz Biotechnology, Dallas, TX, USA). After incubation with secondary horseradish peroxidase–conjugated anti-mouse or anti-rabbit antibodies (Thermo Fisher Scientific), signals were visualized using an enhanced chemiluminescence reagent (ECL) (Millipore, Billerica, MA, USA) in a LAS-4000 lumino-image analyzer system (Fujifilm, Tokyo, Japan).

### Cell proliferation assay

Cell proliferation was determined by means of the Cell Counting Kit-8 (CCK-8) assay (Dojindo Laboratories, Kumamoto, Japan) or BrdU incorporation assay (Cell Signaling Technology), according to the manufacturer’s protocol. Briefly, cells were seeded on 96-well culture plates (0.5×10^4^ cells/well) with 100 μL of growth culture media. After 24 h, the cells were changed to 2% FBS-containing media and cultured for another 24 h. Cells were treated with the indicated amount of FGF2 or were infected with Ad-COUP-TFII, as previously described. After 24 h (for viral infection, up to 48 h), the CCK-8 reagent (10 μL) was added to each well, and the cells were then incubated at 37°C for 2 h. Absorbance was measured by an ELISA reader (Thermo Fisher Scientific) at a 450-nm wavelength. For the BrdU incorporation assay, cells were seeded on 96-well culture plates (0.5×10^4^ cells/well). After 24 h, the cells were transfected with the indicated siRNAs for 4 h and were then changed to 2% FBS-containing media and cultured for 24 h. Cells were treated with FGF2 with or without the indicated chemical inhibitors in 100 μL of 2% FBS-containing media. After 24 h, 10 μL of 10× BrdU solution was added to each well, and the cells were incubated at 37°C for 2 h. After removal of the culture media, the cells were fixed and incubated with anti-BrdU antibodies for 1 h and were then incubated with HRP-conjugated secondary antibodies for 30 min. Finally, the cells were incubated with a tetramethylbenzidine substrate until color development was sufficient for photometric detection. Absorbance at 450 nm was measured by an ELISA reader (Thermo Fisher Scientific).

### Osteoblast differentiation, alkaline phosphatase staining, and alizarin red staining

Cells were seeded on 48-well culture plates (4×10^4^ cells/well) and were treated with FGF2 with or without U0126 every other day for 4 days. After removing FGF2-containing media, cells were cultured with osteogenic media (OM) (50 μg/mL of ascorbic acid, 5 mM of β-glycerophosphate, and 100 ng/mL of BMP2). The induction media was changed every 2 days. For alkaline phosphatase (ALP) enzyme staining, cells were fixed with 4% formaldehyde (Sigma-Aldrich) for 15 min and then treated with a BCIP/NBT solution (Sigma-Aldrich) for 30 min. To evaluate mineralization, cells were fixed with 70% cold ethanol and then treated with 40 mM of alizarin red (Sigma-Aldrich) solution (pH 4.2) for 30 min. The stained culture plates were scanned with an Epson Perfection V700 (Epson Korea, Seoul, South Korea), and the stained cells were observed via optical microscopy (magnification: 50×, Leica Microsystems, Wetzlar, Germany). For quantitative analysis, the alizarin red stain was extracted with 10% (w/v) cetylpyridinium chloride in 10 mM of sodium phosphate (pH 7.0) for 15 min and fluorescence was quantified by measuring absorbance at 540 nm using an ELISA reader (Thermo Fisher Scientific).

## Results

### FGF2 is an extracellular factor regulating COUP-TFII expression in mesenchymal cells

**Fig 1:**
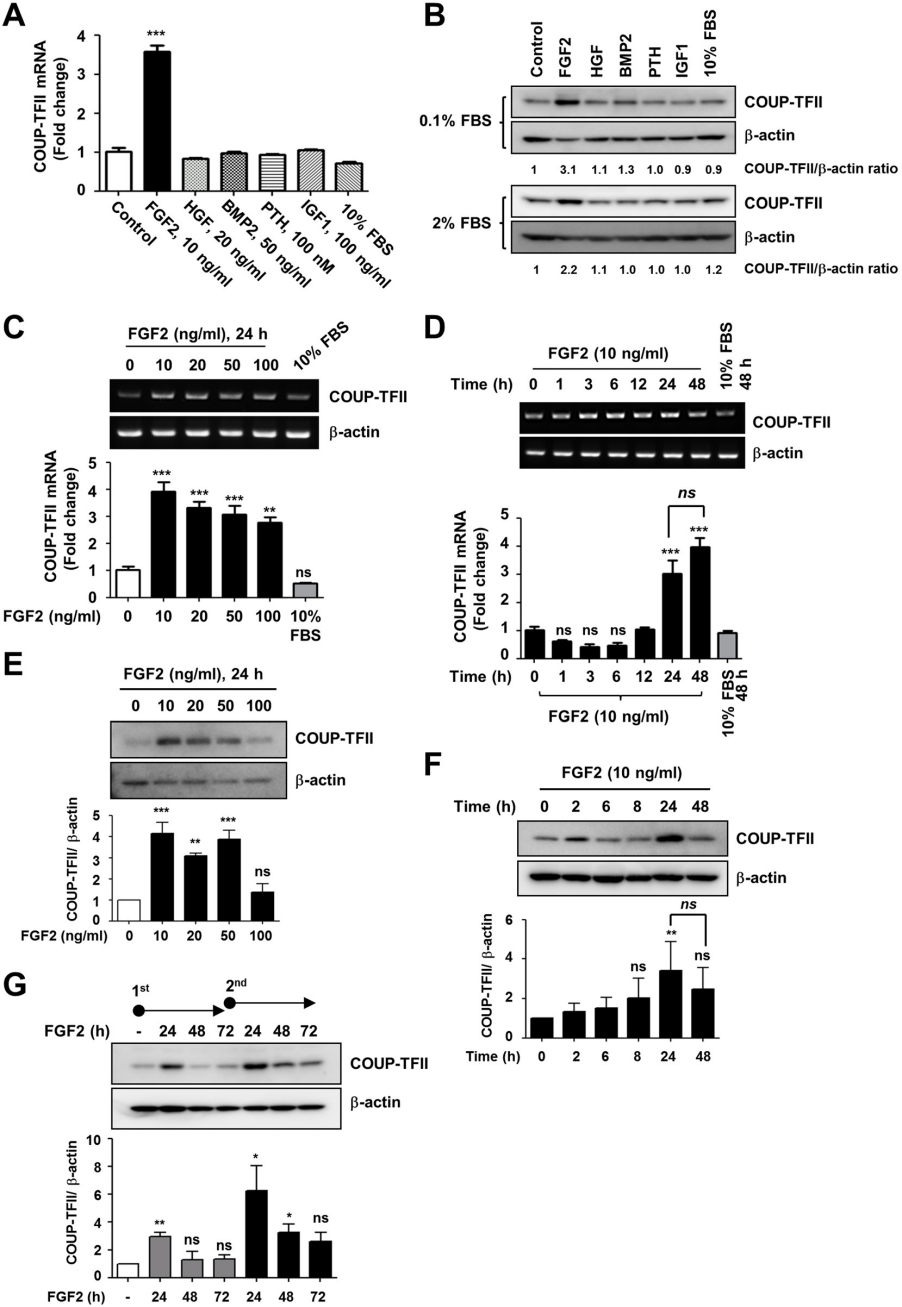
FGF2 induces COUP-TFII expression in C3H10T1/2 cells. (A) C3H10T1/2 cells were cultured in 0.1% FBS-containing DMEM for 24 h and then were treated with the indicated amounts of several extracellular factors. After 24 h, the cells were harvested, and the expression of COUP-TFII was analyzed by real-time RT-PCR. Relative COUP-TFII expression was calculated after normalization to β-actin. (B) C3H10T1/2 cells were cultured in 0.1% or 2% FBS-containing DMEM for 24 h with several extracellular factors, as in panel A. Cell lysates were applied to immunoblotting to analyze COUP-TFII protein levels. The level of β-actin was analyzed as a loading control. Numbers below gel images represent the normalized value of relative COUP-TFII levels. (C, E) After the cells were treated with the indicated amounts of FGF2 for 24 h in the same conditions as in panel A, the expression of COUP-TFII was analyzed by conventional RT-PCR analysis (upper panel) and real-time RT-PCR (lower panel), and by immunoblot analysis. (D, F) Cells were incubated with 10 ng/mL of FGF2 for the indicated time period, cell lysates were prepared, and the expression of COUP-TFII was analyzed as in panels C and E. (G) Effects of repeat treatment of FGF2 on COUP-TFII expression. C3H10T1/2 cells were incubated with 10 ng/mL of FGF2 and were then harvested at the indicated time points to undergo immunoblot analysis. The cells were pre-exposed to 10 ng/mL of FGF2 for first 72 h and then re-exposed (indicated as ball-nocks). COUP-TFII expression was analyzed by means of immunoblot analysis. (A-D) Values for the relative expression of the COUP-TFII gene are expressed as the mean ± SEM of a triplicate reaction of one representative experiment. All experiments were repeated three times. Statistical analysis was performed by ANOVA followed by the Tukey post hoc test. (E-G) Immunoblot bands were quantified by densitometry using Science Lab Image Gauge version 3.0 software (Fujifilm), and the ratio of COUP-TFII/β-actin was determined. Data shown are representative of three independent experiments, and the values are expressed as the mean ± SD of three independent experiments. Statistical analysis was performed by ANOVA with the Bonferroni post hoc test. * p<0.01; ** *p*<0.01; *** *p*<0.001 vs. control.

To identify extracellular factors that are involved in COUP-TFII expression, we analyzed the effect of several growth factors and hormones, known to be major cytokines involved in mesenchymal stem cell functions, on COUP-TFII gene expression in C3H10T1/2 cells. As shown in Fig 1A and 1B, COUP-TFII expression was induced exclusively by FGF2. The ability of FGF2 to induce COUP-TFII expression was maximal at a concentration of 10 ng/mL and gradually decreased with increasing FGF2 concentration (Fig 1C and 1E). Treatment with 10 ng/mL FGF2 significantly induced COUP-TFII expression after 24 h, after which point the expression of COUP-TFII protein tended to slightly decrease rather than increase (Fig 1D, 1F and 1G). Repeat treatment with 10 ng/mL FGF2 after 72 h also induced COUP-TFII expression again, even more so than did the first exposure (Fig 1G). These results suggest that FGF2 may be an extracellular factor that induces COUP-TFII expression in mesenchymal cells.

### Induction of COUP-TFII expression by FGF2 involves MEK1/2 signaling

**Fig 2:**
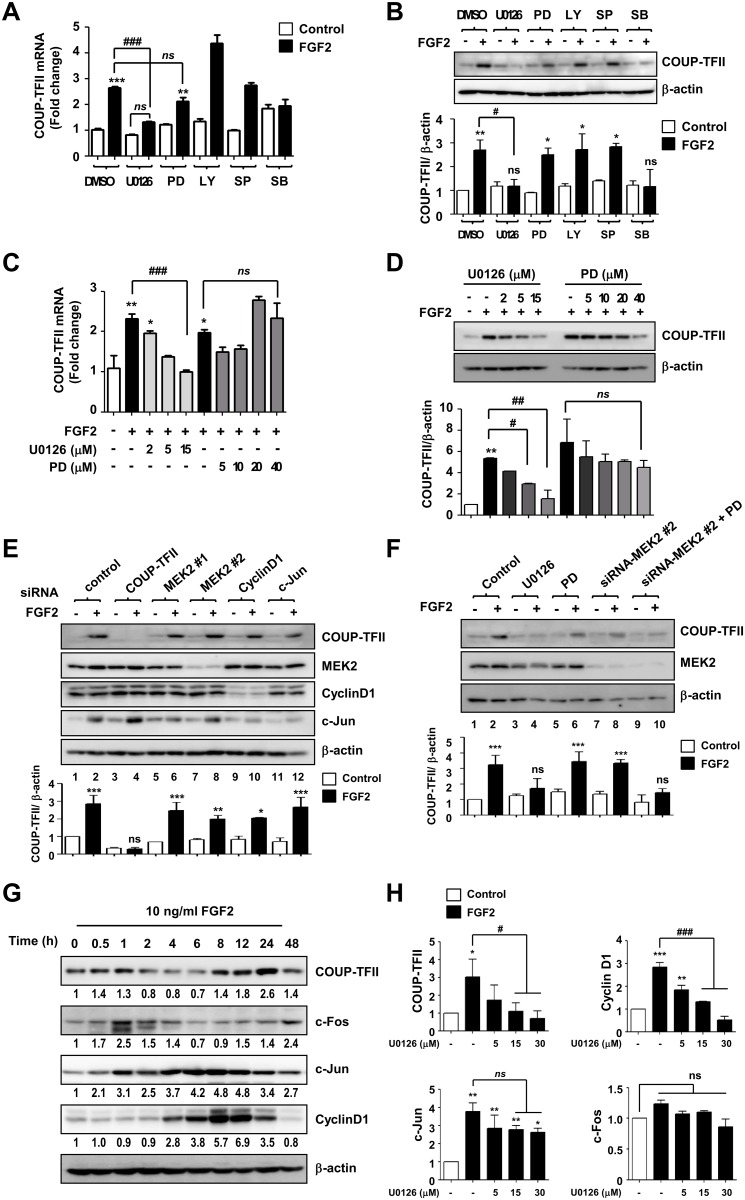
FGF2-induced COUP-TFII expression mediates the MEK1/2 signaling pathway. (A, B) C3H10T1/2 cells were pretreated with DMSO, 5 μM of U0126, 10 μM of PD98059 (PD), 5 μM of LY294002 (LY), 10 μM of SP600125 (SP), or 10 μM of SB202190 (SB) for 30 min, and then FGF2 was added at a concentration of 10 ng/mL. After 24 h, the expression of COUP-TFII was analyzed by means of real-time RT-PCR (A) and immunoblot analysis (B). Relative COUP-TFII expression was calculated after normalization to β-actin (A). (C, D) FGF2 induction of COUP-TFII expression was abolished by U0126 but not by PD98059. Cells were pretreated with the indicated amounts of U0126 or PD98059 for 30 min, and then FGF2 was added. After 24 h, cell lysates were prepared, and COUP-TFII expression was analyzed by real-time RT-PCR and by immunoblot analysis. (E, F) MEK1 and MEK2 can induce COUP-TFII expression. C3H10T1/2 cells were transfected with the indicated siRNAs (E). After 24 h, the cells were incubated with FGF2 for 24 h. Cell lysates were analyzed by immunoblotting for the indicated proteins. Cells transfected with the indicated siRNAs were co-treated with FGF2 and compounds (10 μM of U0126 and 20 μM of PD98059) (F), as in (D). The levels of COUP-TFII and MEK2 were analyzed by immunoblot analysis. (G) Time course effect of FGF2 treatment on COUP-TFII, c-Fos, c-Jun, and Cyclin D1 expression. Cells were treated with FGF2 and then harvested at the indicated time points to analyze COUP-TFII, c-Jun, c-Fos, and Cyclin D1 protein levels by immunoblot analysis. The relative protein levels of the indicated proteins were calculated after normalization to β-actin. (H) Cells were prepared as in (D), and COUP-TFII, c-Jun, c-Fos, and Cyclin D1 protein levels were analyzed by immunoblotting, and their relative level was calculated after normalization to the β-actin level. All immunoblot data shown are representative of three independent experiments, and the values are expressed as the mean ± SD of three independent experiments. Statistical analysis was performed by ANOVA with the Bonferroni post hoc test. * *p*<0.05; ** *p*<0.01; *** *p*<0.001 vs. control, # *p*<0.05; ## *p*<0.01; ### *p*<0.001 vs. indicated group.

FGF2 regulates the expression of various genes for maintaining cellular functions through activating its receptors and downstream signaling pathways, such as the PI3K-AKT and MAP kinase (MEK/ERK, JNK, p38) pathways [18]. To investigate the regulatory mechanism involved in COUP-TFII expression by FGF2, we examined COUP-TFII expression following treatment with chemical inhibitors and small interfering RNAs against signaling molecules that act downstream of FGF2. FGF2 treatment consistently increased COUP-TFII expression, and addition of U0126 (a MEK1/2 inhibitor) and SB202190 (a p38 inhibitor) significantly attenuated the FGF2-mediated induction of COUP-TFII expression (Fig 2A and 2B). LY294002 (a PI3K inhibitor), SP600125 (a JNK inhibitor), and PD98059 (a specific inhibitor of MEK1) did not inhibit the actions of FGF2. The inhibitory effect of U0126 on FGF2-mediated COUP-TFII expression was dose-dependent, and PD98059 had no effect on COUP-TFII expression (Fig 2C and 2D), implying that MEK2 may be more important than MEK1 for COUP-TFII induction. Unexpectedly, we observed that FGF2 continues to induce COUP-TFII expression in MEK2-silenced cells (Fig 2E, lanes 7 and 8). Moreover, the inhibitory effect of PD98059 on COUP-TFII expression was found to emerge only in MEK2-silenced cells (Fig 2F, lanes 6 and 10). These results suggest that activation of either MEK1 or MEK2 is sufficient to mediate the effect of FGF2 on COUP-TFII induction.

Given that COUP-TFII mRNA was induced 24 h after FGF2 treatment (Fig 1D), we hypothesized that the expression of FGF2-induced transcription factors might precede COUP-TFII expression. The transcription factors c-Jun, c-Fos, and cyclin D1 were identified to be induced by FGF2 via the MEK/ERK pathway [19–21]. To identify the transcription factor which responsible for COUP-TFII induction among them, we analyzed the time course effect of FGF2 on c-Jun, c-Fos, Cyclin D1, and COUP-TFII expression. Consistent with the previous report [22], c-Fos expression was increased rapidly from 30 min after FGF2 stimulation, peaked at 1 hr, and then rapidly returned to baseline (Fig 2G). c-Jun and Cyclin D1 expression started to be increased at the early time point and then sustained up to 24 hr after FGF2 treatment (Fig 2G). In addition, we observed that U0126 inhibits the FGF2-induced expression of COUP-TFII and Cyclin D1 in a dose-dependent manner, but not c-Jun expression (Fig 2H). Based on these results, we assumed that COUP-TFII induction is likely to be related to the elevation of Cyclin D1, rather than c-Jun or c-Fos. However, silencing of cyclin D1 or c-Jun by siRNAs did not affect FGF2-induced COUP-TFII expression (Fig 2E, lanes 9–12). Although we could not identify specific transcription factor that induced COUP-TFII expression in the current study, our findings suggest that the expression of COUP-TFII is regulated by the MEK1/2 pathway in a c-Jun- and Cyclin D1-independent manner.

### COUP-TFII induction is not required for FGF2-induced cell proliferation

**Fig 3:**
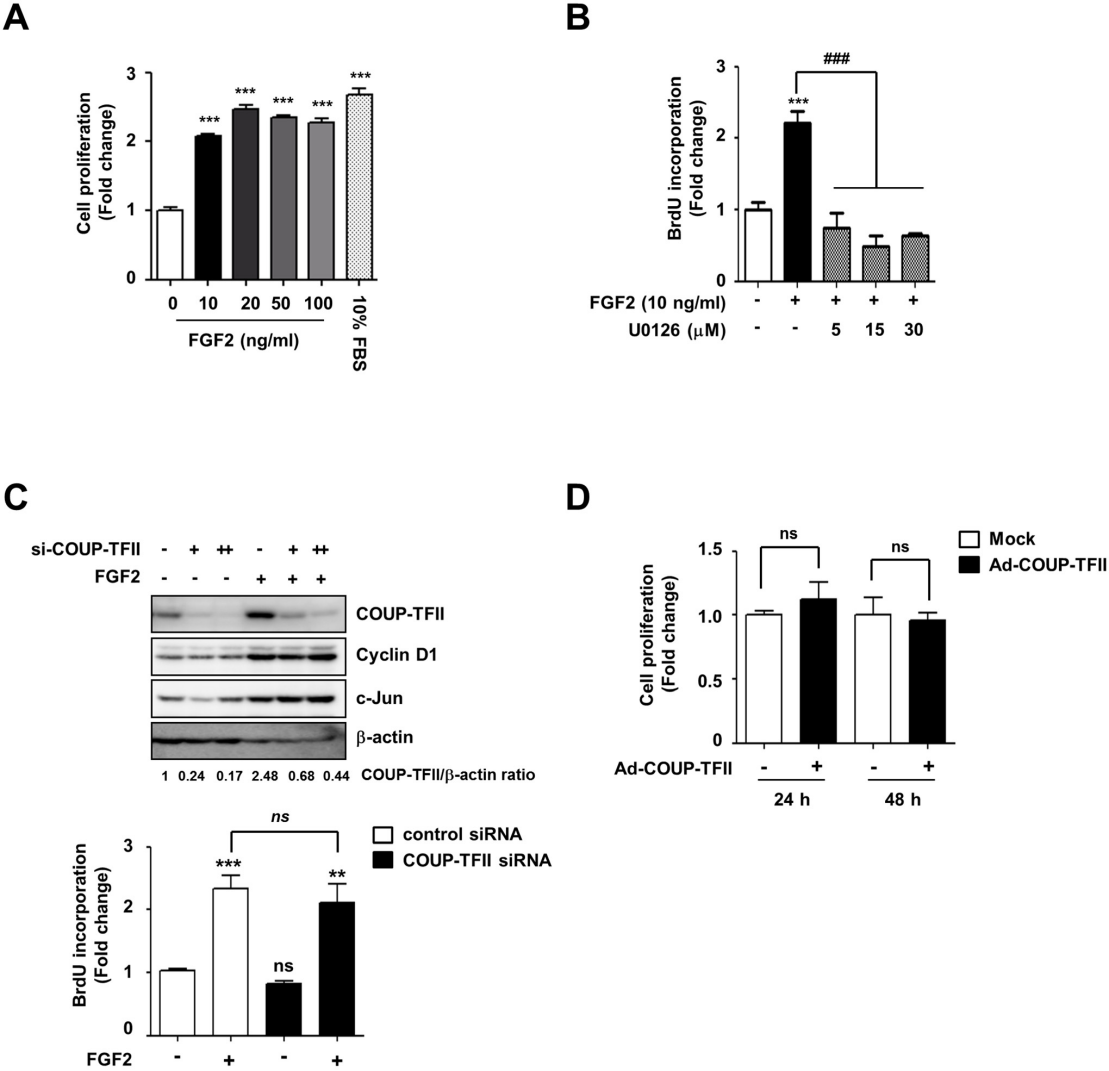
COUP-TFII is not involved in FGF2-mediated cell proliferation. (A) FGF2 increases C3H10T1/2 cell proliferation. Cells were starved with 2% FBS-containing DMEM for 24 h and then were exposed to various concentrations of FGF2 (10–100 ng/mL) for 24 h. Cell proliferation was measured with a CCK-8 assay, with 10% FBS used as a positive control. (B) FGF2-inducd cell proliferation is mediated by the MEK1/2 pathway. Serum-deprived cells were incubated with 10 ng/mL of FGF2 in the presence or absence of U0126. After 24 h, cell proliferation was measured by the BrdU incorporation assay. (C, D) COUP-TFII does not affect C3H10T1/2 cell proliferation. Cells transfected with si-COUP-TFII (+, 70 nM; ++, 100 nM) were treated with FGF2 (C), as in panel A. After 24 h, cell proliferation was measured by the BrdU incorporation assay. The levels of COUP-TFII, Cyclin D1, and c-Jun were determined by immunoblot assay. Numbers below gel images represent the normalized value of relative COUP-TFII levels. (D) Cells infected with Ad-COUP-TFII (+, 50 multiplicity of infection) or Mock (Ad-GFP, 50 multiplicity of infection) were cultured in 2% FBS-containing DMEM for the time indicated, and cell proliferation was measured by CCK-8 assay. Representative data from three independent experiments are shown. All cell proliferation data are expressed as mean ± SEM of triplicate reactions of one representative experiment. Statistical analysis was performed by ANOVA followed by the Tukey post hoc test. ** *p*<0.01; *** *p*<0.001 vs. control (first bar); ### *p*<0.001 vs. indicated group.

FGF2 has been shown to enhance mesenchymal stem cell proliferation through activation of the MAPK signaling cascade [18, 20, 23]. Meanwhile, COUP-TFII has been reported to enhance breast and endothelial cell proliferation with increases in the expression of Cyclin D1 and E2F1, two major G1/S transition phase regulators [24, 25]. Given the mitogenic function of FGF2 and COUP-TFII, we also examined whether COUP-TFII is involved in FGF2-mediated mesenchymal cell proliferation. FGF2 treatment increased cell proliferation by approximately two-fold, and addition of U0126 halted the FGF2-stimulated cell proliferation (Fig 3A and 3B). Silencing of COUP-TFII did not affect FGF2-induced expression of Cyclin D1, a key transcription factor for cell proliferation (Fig 3C). More importantly, COUP-TFII silencing did not alter FGF2-stimulated cell proliferation (Fig 3C, compare the second with the fourth bars in the graph). Overexpression of COUP-TFII with the COUP-TFII-GFP adenovirus did not change basal cell proliferation activity (Fig 3D). These results suggest that the FGF2/COUP-TFII pathway may not be involved in FGF2 stimulation of cell proliferation.

### FGF2-primed cells exhibit reduced osteogenic differentiation potential owing to COUP-TFII induction

**Fig 4:**
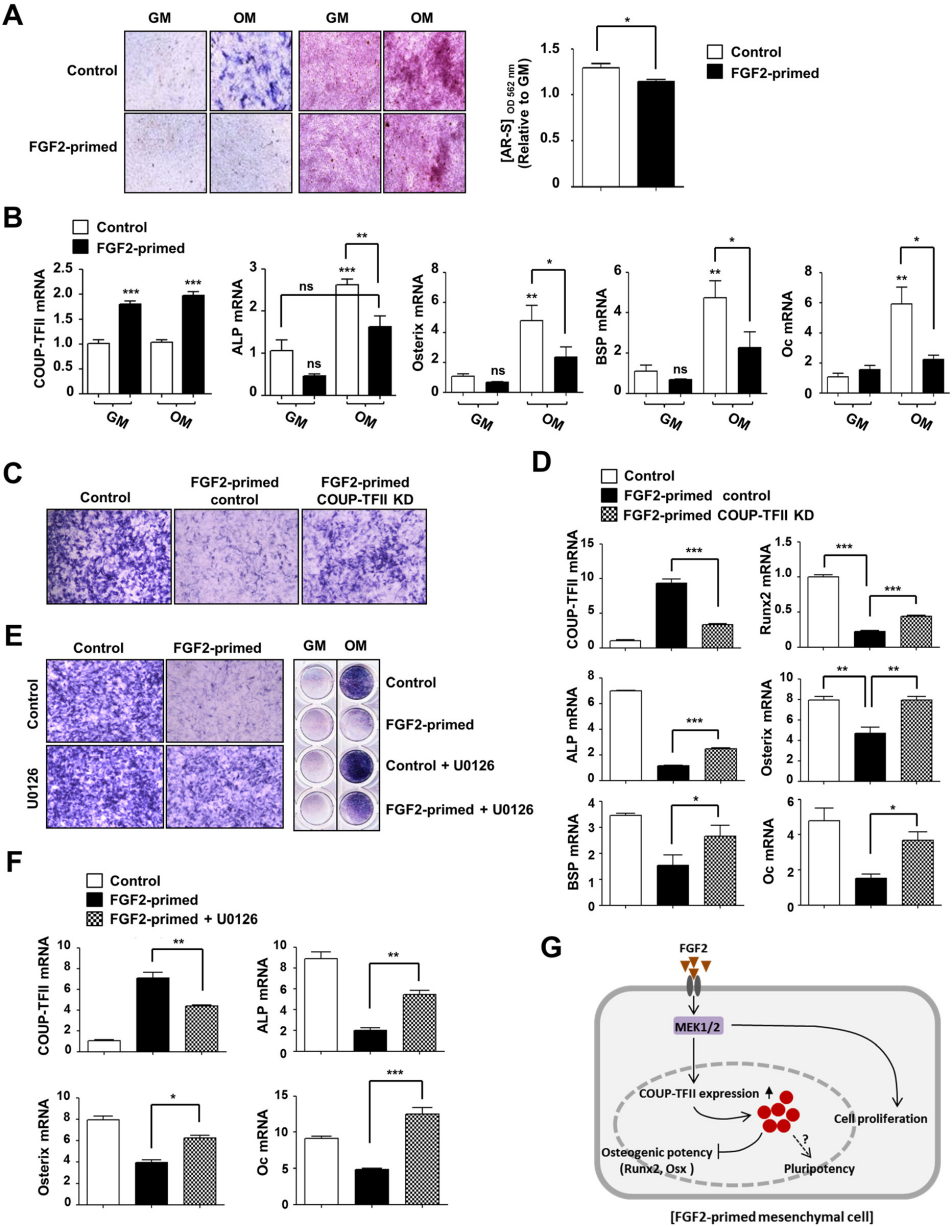
COUP-TFII induction by FGF2 priming leads to a reduction in osteodifferentiation potential. (A, B) Pre-exposure to FGF2 prior to differentiation stimuli inhibits osteoblast differentiation of C3H10T1/2 cells. (A) Cells were treated with 10 ng/mL of FGF2 every other day for 4 days. After removing FGF2-containing media, cells were incubated with osteogenic media (OM) (50 μg/mL of ascorbic acid, 5 mM of β-glycerophosphate, and 100 ng/mL of BMP2). Cells were stained for alkaline phosphatase activity after 5 days of differentiation (left, ALP staining), and were subjected to alizarin red staining after 10 days of differentiation (right, AR staining). The bar graph shows the relative intensity of AR staining. Cells stained with AR were incubated in 10% cetylpyridinium chloride, and staining was quantified at 562 nm. The ratio of OM/GM was determined. (B) After cells were prepared as in panel A, cells were harvested on day 5 for COUP-TFII, ALP, and Osterix, and on day 10 after differentiation for BSP and osteocalcin (Oc). Total RNA was isolated and subjected to real-time RT-PCR. (C-F) Blocking of COUP-TFII induction abolished the anti-osteogenic effect of FGF2 priming. (C) After COUP-TFII–silenced cells were pretreated with FGF2 as in panel A, osteogenic differentiation was induced for 4 days. Alkaline phosphatase activity in the differentiated cells was analyzed by ALP staining. Control, non-FGF2 treated and control siRNAs-transfected cells; FGF2-primed control, FGF2-treated and control siRNA-transfected cell. (D) Osteogenic differentiated cells were harvested and subjected to real-time RT-PCR to analyze expression levels of Osterix (on day 4), ALP (on day 2), BSP and Oc (on day 10). The relative expression levels of COUP-TFII and Runx2 were analyzed on day 0 (that is, before the onset of differentiation). (E) Cells were pretreated with FGF2 as in panel A in the presence or absence of U0126, and the cells then underwent osteogenic differentiation for 4 days. Alkaline phosphatase activity was determined by ALP staining, and magnified images of the differentiated cells are representative of the relevant wells (left). (F) Before the onset of differentiation (day 0), the cells were harvested for analysis of COUP-TFII levels. The relative expression levels of ALP (on day 2), Osterix (on day 4), and Oc (on day 10) were determined. Representative data from three independent experiments are shown. Values for the relative expression of the indicated genes are expressed as the mean ± SEM of triplicate reactions in one representative experiment. Statistical analysis was performed by ANOVA followed by the Tukey post hoc test. * *p*<0.05; ** *p*<0.01; *** *p*<0.001. (G) Working model for the role of overexpressed COUP-TFII in the FGF2-primed mesenchymal cells. FGF2 priming in uncommitted mesenchymal cells induces COUP-TFII expression via the MEK1/2 pathway and it might bring about low osteogenic potential and high pluripotency.

COUP-TFII is known to be a major negative regulator of osteogenesis [3, 4, 7]. FGF2 was reported to be involved in the multilineage differentiation of mesenchymal cells [26, 27]. Although FGF2 is implicated in osteogenic differentiation, its role in these processes has been conflicting [28–32]. Given the effect of FGF2 on COUP-TFII induction and the anti-osteogenic function of COUP-TFII, we evaluated the osteogenic differentiation potential of FGF2-primed COUP-TFII–induced mesenchymal cells. C3H10T1/2 cells were cultured with FGF2 (FGF2-primed) or without FGF2 (control) for 4 days and were then incubated with osteogenic medium. The osteogenic differentiation of the FGF2-primed cells was significantly decreased, as evidenced by decreased alkaline phosphatase activity and matrix mineralization (Fig 4A) and confirmed by a reduced expression level of osteogenic marker genes (Fig 4B). To determine whether COUP-TFII induction is related with the decreased osteogenic differentiation of the FGF2-primed cells, we examined the effect of COUP-TFII knock-down (COUP-TFII KD) on the osteogenic differentiation of FGF2-primed cells. COUP-TFII-silenced cells were pre-exposed to FGF2 and were then exposed to osteogenic medium. FGF2-primed COUP-TFII–silenced cells showed attenuated anti-osteogenic phenotypes, as indicated by the restoration of alkaline phosphatase activity and the increased expression of osteogenic marker genes, as compared with the FGF2-primed cells (Fig 4C and 4D). Before the onset of differentiation, FGF2-priming led to decreases in Runx2, Osterix, and ALP expression, which correlated inversely with COUP-TFII levels (Fig 4D). This result implies that the FGF2-induced anti-osteogenic effect may be a result of the COUP-TFII–mediated Runx2 suppression. Likewise, FGF2-mediated inhibition of osteogenic differentiation was abolished by U0126 treatment (Fig 4E and 4F), implying that FGF2-induced COUP-TFII expression but not FGF-mediated cell proliferation is a causative factor in the appearance of the anti-osteogenic differentiation phenotype of the FGF2-primed cells. Taken together, these results suggest that pre-exposure to FGF2 before the onset of differentiation leads mesenchymal cells to have a low osteodifferentiation potential, which is attributable to the induction of COUP-TFII expression.

## Discussion

Since COUP-TFII is recognized as a critical player controlling mesenchymal cell commitment and differentiation, elucidating the signal transduction mechanisms for the regulation of COUP-TFII expression has posed a major challenge to the field of COUP-TFII–mediated pathophysiology. In this study, we demonstrated that COUP-TFII expression is regulated by FGF2, which requires activation of either MEK1 or MEK2. This finding suggests that endogenous COUP-TFII levels may be under the control of the MEK1/ERK1 and MEK2/ERK2 pathways in mesenchymal cells. Given that multiple intracellular signaling pathways are linked to the MEK1/2-ERK1/2 module [33, 34], diverse hormones and extracellular cues that are reportedly linked to this module should be further evaluated as putative physiological ligands for COUP-TFII. We anticipate that this work will provide key information for addressing two important issues: (1) strategies for controlling mesenchymal cell commitment and differentiation in tissue engineering, and (2) identifying the most effective strategy for controlling COUP-TFII–related human diseases [35].

To our knowledge, no physiological ligands for COUP-TFII expression have been identified in uncommitted precursor cells (mesenchymal stem cells). Our study is the first report to identify FGF2 as a specific growth factor that regulates COUP-TFII expression in mesenchymal cells. Fibroblast growth factors (FGFs) are secreted glycoproteins that regulate embryonic development through their involvement in cellular proliferation, differentiation, and cell death [36]. Among the members of the FGF family, FGF2 plays an important role in the expansion and self-renewal of mesenchymal stem cells [19, 20, 23, 37]. We found that FGF2 induction of COUP-TFII expression does not involve mesenchymal cell proliferation (Fig 3), which implies that COUP-TFII is not implicated in FGF2-induced precursor cell expansion. It is one of the important factors that control cellular senescence and cellular apoptosis in fulfilling the need for cell proliferation. FGF2 has also been implicated in suppressing the senescence of human mesenchymal stem cells and enhancing osteoblast survival [38, 39]. Related to this, we are interested in our finding that COUP-TFII expression is downstream of the p38 signaling pathway (Fig 2A and 2B). Since the p38 pathway is largely implicated in cellular stress responses, including inflammation and cell death [40], FGF2 regulation of COUP-TFII expression via the p38 pathway may be involved in cellular senescence and survival. Although we did not examine this hypothesis in the present study, we hope to determine in the near future whether COUP-TFII expression responds to cellular stresses and whether FGF2-induced COUP-TFII plays a role in cellular senescence and survival.

Given that FGF2 induces COUP-TFII expression in mesenchymal cells and pre-osteoblasts (MC3T3-E1 cells) but not in pre-adipocytes (3T3-L1) (S1 Fig), we surmised that FGF2-induced COUP-TFII is largely involved in the commitment to osteogenic lineage determination or osteogenic lineage-specific events. COUP-TFII induction by FGF2 was related to the inhibition of osteogenic differentiation of mesenchymal cells (Fig 4), suggesting that FGF2 has an anti-osteogenic function owing to upregulation of COUP-TFII in these cells. In line with our observation, previous reports showed that FGF2 has an inhibitory effect on osteoblast differentiation by controlling osteogenic gene expression in osteoprogenitor cells and mesenchymal stem cells [29, 41, 42]. In contrast to our observations, there are several reports stating FGF2 plays a positive role in bone formation: FGF2 has been shown to enhance *in vitro* osteogenesis of C3H10T1/2 cells by inducing the expression of TAZ, a Runx2 co-activator [30], and of bone marrow-derived mesenchymal cells [28]. FGF2-null mice were shown to have decreased bone mass and bone formation [43]. Although these differences in observations are still puzzling, it has been accepted that the opposite effect of FGF2 on *in vitro* osteogenesis may be the result of differing experimental conditions (e.g. cell type, incubation periods, the concentration of FGF2, and the differentiation protocol). More importantly, co-incubation of FGF2 with osteogenic media was shown to exert an anti-osteogenic effect in adipose-derived stem cells [32], while pre-exposure to FGF2 before the onset of differentiation was found to enhance the osteodifferentiation potential of these cells [31]. These findings imply that the duration and timing of FGF2 exposure are important factor in determining mesenchymal cell fates. We also observed enhanced osteoblast differentiation of C3H10T1/2 cells when FGF2 was co-treated with osteogenic differentiation media (S2 Fig). On the other hand, FGF2 pretreatment before the onset of differentiation inhibited osteogenic differentiation (Fig 4C–4F). These observations support our assumption that the timing of FGF2 exposure is an important parameter in mesenchymal cell fate determination. Relatedly, a recent study showed that FGF2 has biphasic effects on adipogenesis depending on its concentration (as a negative factor at high concentrations and as a positive factor at low concentrations) in human adipose-derived stem cells [44], which points to the importance of applying the proper concentration of FGF2. Considering these findings and the fact that FGF2 exhibits differentiation stage–specific effects on cellular differentiation [45], the role of FGF2 as an osteogenic regulator should be re-evaluated carefully further.

When precursor cells are pre-exposed to FGF2 and it is removed before the onset of differentiation, the osteogenic differentiation process is delayed owing to the presence of high levels of COUP-TFII at the initiation stage. Nevertheless, we could not fully understand why pre-exposure to FGF2 has an anti-osteogenic effect on precursor cells. A recent study found that pre-exposure to FGF2 plays a role in preventing the loss of precursor cell characteristics and differentiation potential by inducing the expression of self-renewal regulators [46]. COUP-TFII is also implicated in embryonic stem cell pluripotency and reprogramming [15, 16]. Based on these reports, we postulate a scenario in which COUP-TFII induction by FGF2 plays a role in maintaining the potency of precursor cells through delaying the initiation of osteoblast differentiation. Given the fact that COUP-TFIIs are predominantly expressed in uncommitted precursor cells, it will be important to examine whether FGF2-induced COUP-TFII is involved in maintaining the “stemness” of precursor cells via transcriptional regulation of these self-renewal factors. Our ongoing study related to this issue will soon provide insights into how it is possible to prevent the loss of progenitor cell properties and why COUP-TFII expression is high in uncommitted precursor cells.

Collectively, we hypothesize that FGF2 may be a strong extracellular inducer of COUP-TFII expression, and that FGF2 determination of mesenchymal cells fates and pluripotency may mediate the nuclear receptor COUP-TFII (Fig 4G). These findings could be applied to develop a new strategy for tissue regeneration using mesenchymal stem cells.

## Supporting Information

S1 Fig FGF2 induces COUP-TFII expression in C3H10T1/2 and MC3T3-E1 cells, but not in 3T3-L1 cells. (A) C3H10T1/2, MC3T3-E1, and 3T3-L1 cells were serum-deprived with 0.1% FBS-containing DMEM for 24 h and were then incubated with 10 ng/mL of FGF2 in 2% FBS-containing media for the time period indicated. Cells were prepared, and the COUP-TFII mRNA level was determined by conventional RT-PCR analysis. (B) Cells were treated with FGF2 as in panel A. After a 24 h treatment, COUP-TFII expression was analyzed by means of real-time RT-PCR. Relative COUP-TFII expression was calculated after normalization to β-actin. Values for the relative expression of COUP-TFII gene were expressed as the mean ± SEM of triplicate reaction of one representative experiment. All experiments were repeated three times. Statistical analysis was performed by ANOVA followed by the Tukey post hoc test. *** *p*<0.001.
(TIF)

S2 Fig Co-treatment with FGF2 and osteogenic media enhances osteogenic differentiation of C3H10T1/2 cells. Cells were differentiated into osteoblasts in the absence (OM) or presence of 10 ng/mL of FGF2 (OM + FGF2). Total RNA was isolated and subjected to real-time RT-PCR. Relative expression levels of Osterix, BSP, and osteocalcin (Oc) were determined after normalization to β-actin. Values for the relative expression of COUP-TFII gene were expressed as the mean ± SEM of triplicate reaction of one representative experiment. All experiments were repeated three times. Statistical analysis was performed by ANOVA followed by the Tukey post hoc test. *** *p*<0.001. OM = osteogenic medium (10% FBS-DMEM containing 100 ng/mL of BMP2, 50 μg/mL of ascorbic acid, and 5 mM of β-glycerophosphate).
(TIF)
