# Supplementary figures and images for: FGF2 Stimulates COUP-TFII Expression via the MEK1/2 Pathway to Inhibit Osteoblast Differentiation in C3H10T1/2 Cells
Source: PLoS One. 2016 Jul 12;11(7):e0159234. doi: 10.1371/journal.pone.0159234 (PMC4942136; doi:10.1371/journal.pone.0159234)

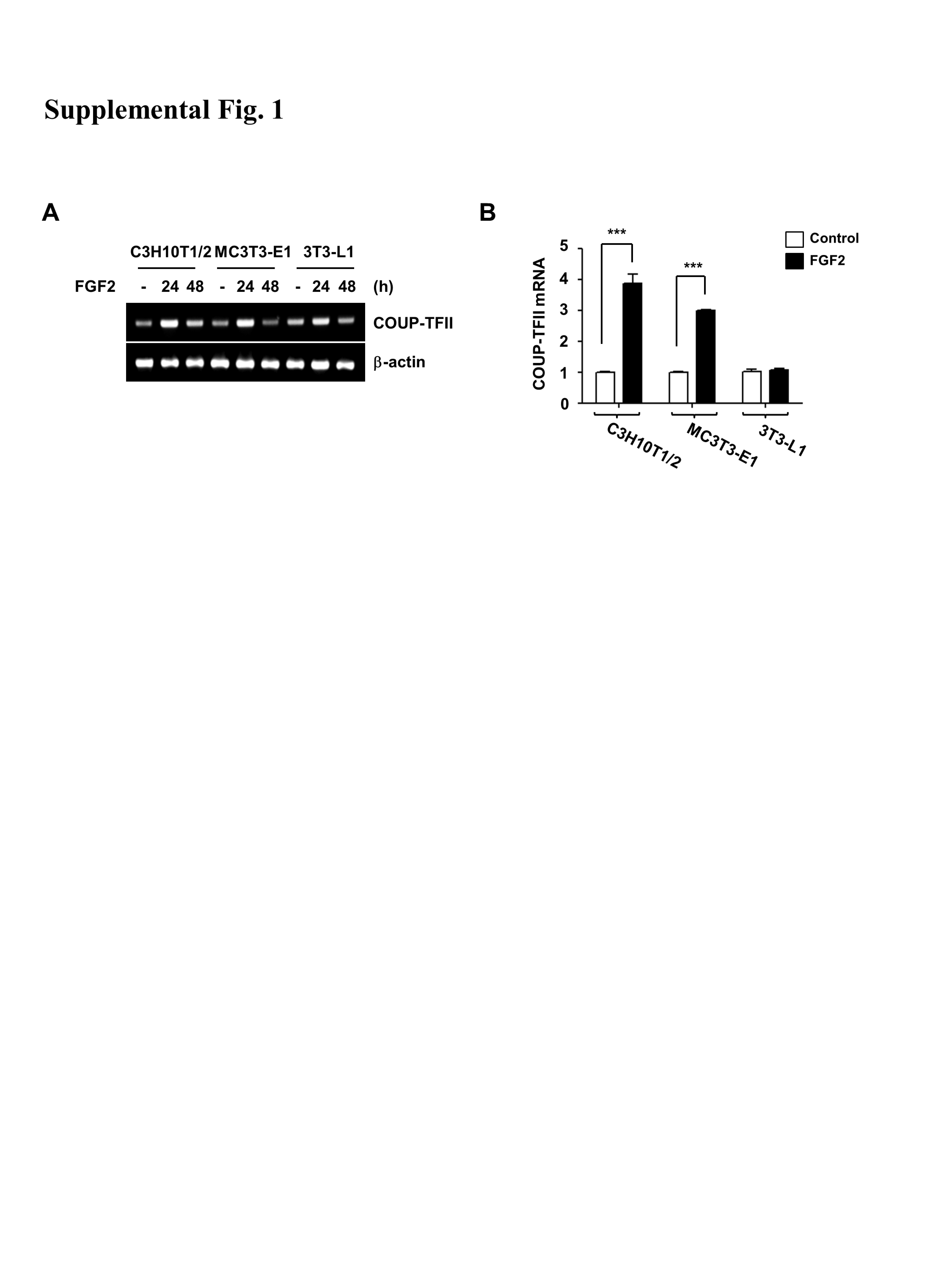

Supplement: S1 Fig — (A) C3H10T1/2, MC3T3-E1, and 3T3-L1 cells were serum-deprived with 0.1% FBS-containing DMEM for 24 h and were then incubated with 10 ng/mL of FGF2 in 2% FBS-containing media for the time period indicated. Cells were prepared, and the COUP-TFII mRNA level was determined by conventional RT-PCR analysis. (B) Cells were treated with FGF2 as in panel A. After a 24 h treatment, COUP-TFII expression was analyzed by means of real-time RT-PCR. Relative COUP-TFII expression was calculated after normalization to β-actin. Values for the relative expression of COUP-TFII gene were expressed as the mean ± SEM of triplicate reaction of one representative experiment. All experiments were repeated three times. Statistical analysis was performed by ANOVA followed by the Tukey post hoc test. *** p<0.001. (TIF) [file pone.0159234.s001.tif]

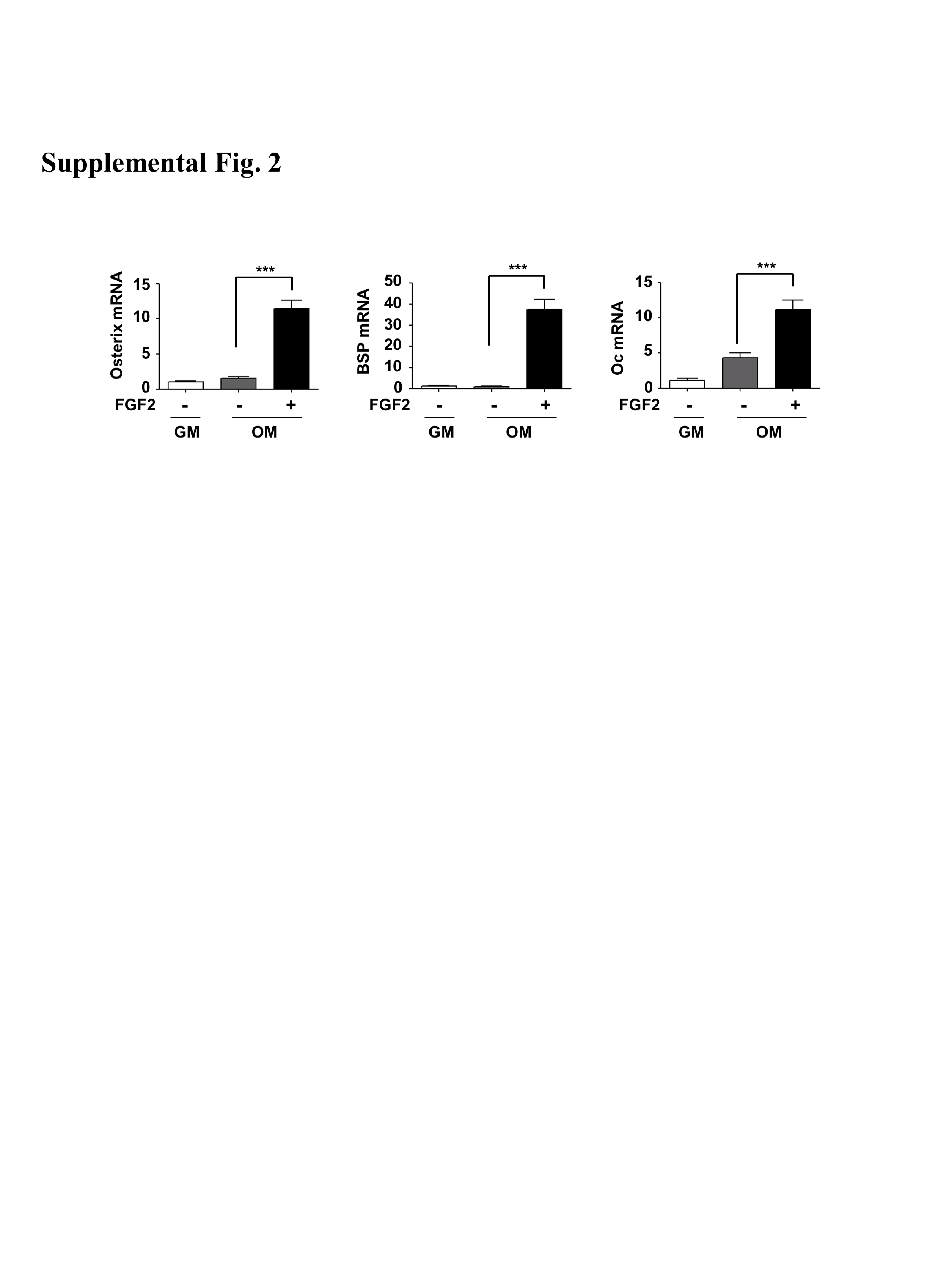

Supplement: S2 Fig — Cells were differentiated into osteoblasts in the absence (OM) or presence of 10 ng/mL of FGF2 (OM + FGF2). Total RNA was isolated and subjected to real-time RT-PCR. Relative expression levels of Osterix, BSP, and osteocalcin (Oc) were determined after normalization to β-actin. Values for the relative expression of COUP-TFII gene were expressed as the mean ± SEM of triplicate reaction of one representative experiment. All experiments were repeated three times. Statistical analysis was performed by ANOVA followed by the Tukey post hoc test. *** p<0.001. OM = osteogenic medium (10% FBS-DMEM containing 100 ng/mL of BMP2, 50 μg/mL of ascorbic acid, and 5 mM of β-glycerophosphate). (TIF) [file pone.0159234.s002.tif]
